# Dissection of Host Cell Signal Transduction during *Acinetobacter baumannii* – Triggered Inflammatory Response

**DOI:** 10.1371/journal.pone.0010033

**Published:** 2010-04-07

**Authors:** Catalina March, Verónica Regueiro, Enrique Llobet, David Moranta, Pau Morey, Junkal Garmendia, José A. Bengoechea

**Affiliations:** 1 Program Infection and Immunity, Fundació Caubet-CIMERA Illes Balears, Bunyola, Spain; 2 Area Molecular Basis of Microbial Pathogenesis, Centro de Investigación Biomédica en Red Enfermedades Respiratorias, Bunyola, Spain; 3 Instituto de Agrobiotecnología, Pamplona, Spain; 4 Área de Microbiología, Facultad Biología, Universitat Illes Balears, Palma Mallorca, Spain; 5 Consejo Superior de Investigaciones Científicas (CSIC), Madrid, Spain; Universidad Nacional, Costa Rica

## Abstract

Infected airway epithelial cells up-regulate the expression of chemokines, chiefly IL-8, and antimicrobial molecules including β-defensins (BD). *Acinetobacter baumannii* is a cause of hospital-acquired pneumonia. We examined whether *A. baumannii* induced the expressions of IL-8 and BD2 by airway epithelial cells and the receptors implicated in bacterial detection. A549 and human primary airway cells released IL-8 upon infection. *A. baumannii*-infected cells also increased the expression of BD2 which killed *A. baummannii* strains. IL-8 induction was via NF-κB and mitogen-activated kinases p38 and p44/42-dependent pathways. *A. baumannii* engaged Toll-like receptor (TLR) 2 and TLR4 pathways and A549 cells could use soluble CD14 as TLRs co-receptor. *A. baumannii* lipopolysaccharide stimulated IL-8 release by A549 cells and sCD14 facilitated the recognition of the lipopolysaccharide. Mass spectrometry analysis revealed that *A. baumannii* lipid A structure matches those with endotoxic potential. These results demonstrate that airway epithelial cells produce mediators important for *A. baumannii* clearance.

## Introduction

*Acinetobacter baumannii* has been implicated in a variety of nosocomial infections being the pulmonary compartment the predominant site of infection [1]. The crude mortality of *A. baumannii* ventilator associated pneumonias is as high as 75% [2]. *A. baumannii* infections are extremely difficult to treat because of the widespread resistance of these bacteria to the major groups of antibiotics [1]. Despite the clinical relevance of *A. baumannii* there is little information on the virulence factors expressed by this pathogen [1].

Inflammatory response plays an essential role in lung defence against pathogens. This response includes, among others, the production of pro-inflammatory and chemoattractant cytokines [3]–[8]. In turn, chemokines are required for the recruitment of neutrophils into airways which is a common histological finding in patients with pneumonia, independently of the infecting microorganism [9]. Current evidence shows that IL-8 targets neutrophils to sites of attack through its chemoattractant and activating properties [10]. It has become evident that airway epithelial cells play a pivotal role in lung defense by detecting pathogens which leads to the expression of co-stimulatory molecules and the release of cytokines and chemokines that influence airway inflammation [11]. Airway epithelial cells also produce antimicrobial molecules being β-defensin (BD) 2 one of the most studied defensin [12], [13]. The expression of BD2 by airway epithelial cells is induced by cytokines or by the presence of pathogens [14]–[17] and it has been shown that BD2 levels increase several folds in lung during pneumonia [18].

A wealth of evidence indicates that members of the Toll-like receptor (TLR) family are key receptors signaling the presence of pathogens to airway epithelial cells [11] being TLR2 and TLR4 the most extensively studied TLRs. While TLR4 seems to be mainly involved in the detection of lipopolysaccharide (LPS), TLR2 responds to a variety of products from gram positive bacteria [19]–[21].

It has been recently demonstrated that the release of pro-inflammatory mediators, chemokines and the recruitment of large number of neutrophils are essential for the clearance of *A. baumannii* within the lungs [22]–[24]. Furthermore, evidence shows that TLR4-dependent signaling plays an important role in sensing of *A. baumannii in vivo* because TLR4-deficient mice challenged with *A. baumannii* displayed an impaired lung inflammatory response [22]. Taken into account the important role of airway epithelial cells in orchestrating lung immunity, we hypothesized that airway epithelial cells would play a key role in sensing *A. baumannii* infections thereby leading to production of mediators necessary for the clearance of *A. baumannii*. In this study, we provide insight into the signaling pathways and receptors involved in sensing *A. baumannii* by human airway epithelial cells.

## Materials and Methods

### Bacterial strains, growth conditions and reagents

Non clonally related *A. baumannii* clinical isolates 1514, 670, 1064 and 1327, all of them resistant to cefotaxime, ceftazidime, imipenem, amoxicillin, amikacin, piperacillin-tazobactam, doxycycline, and colistin sulphate, were from the collection of *A. baumannii* strains at the “Virgen del Rocío” University Hospital, (Seville, Spain). *A. baumannii* strain ATCC 17978 was obtained from the ATCC. Bacteria were grown in Luria-Bertani (LB) at 37°C.

Caffeic acid phenethyl ester (CAPE), an NF-κB inhibitor, and SB203580, a p38 MAPK inhibitor, were purchased from Sigma whereas U0126, a p44/42 MAPK inhibitor, and SP600125, a JNK inhibitor, were purchased from Calbiochem. Purified recombinant human sCD14 was purchased from R&D Systems.

### Cell culture and infections

Monolayers of human lung carcinoma cells (A549, ATCC CCL185) and primary human airway epithelial cells (NHBE, Lonza) were grown as previously described [25]. 18 h before infection A549 cells were serum starved. Prior to the experiment, A549 and NHBE cells were washed twice with PBS. Overnight-grown bacteria were subcultured and grown to exponential phase, harvested by centrifugation (2000×*g*, 20 min, 5°C) and resuspended in PBS. Infections were performed using a multiplicity of infection of 100 bacteria per cell. Cell viability was assessed by trypan blue dye exclusion and it was >95% even after 8 h post infection or after treatments with either CAPE, SB203580, U0126 or SP600125 for the times or concentrations used in this study.

### IL-8 stimulation assay

Epithelial monolayers were infected for different time points. Supernatants were removed from the wells, cell debris removed by centrifugation, and samples were frozen at –80°C. IL-8 in the supernatants was determined by a commercial ELISA (Endogen) with a sensitivity <2 pg/ml. Experiments were run in duplicate and repeated at least three times.

### Immunoblotting

Proteins from lysed cells (15 µg) were separated by 10% SDS-PAGE, electrotransferred to nitrocellulose membrane and blocked with 4% skimmed milk in PBS. Immunostaining for IκBα was performed with polyclonal rabbit anti- IκBα antibody (Santa Cruz Biotecnology) whereas immunostainings to assess phosphorylation of NF-κB p65, mitogen-activated protein kinases (MAPKs) p38, p44/42 and JNK were performed with polyclonal rabbit anti-phospho p65, anti-phospho-p38 antibody, anti-phospho-p44/42 and anti-phospho-JNK antibodies respectively (Cell Signaling). Immunoreactive bands were visualized by incubation with swine anti-rabbit immunoglobulins conjugated to horseradish peroxidase (Dako P0217) using the SuperSignal West-dura system (Pierce). Blots were reprobed with polyclonal antibody anti human tubulin to control that equal amounts of proteins were loaded in each lane.

### Nuclear cytoplasmic fractionation

Nuclear and cytosolic proteins were extracted from approximately 5×10^6^ cells as previously described [25]. Briefly, A549 cells were washed with ice-cold PBS and suspended in hypotonic buffer (10 mM HEPES, 10 mM KCl, 2 mM MgCl_2_, 1 mM DTT, 0.1 mM EDTA, 0.2 mM NaF, 0.2 mM Na_3_VO_4_, 1 µg/ml leupeptin, 0.4 mM PMSF). The cells were left on ice for 15 min and cytosolic preparation was made by addition of Nonidet P-40 (final concentration of 0.1%) followed by centrifugation (6500 rpm, 3 min, 4°C). The supernatant was collected as the cytosolic fraction. The remaining pellet was resuspended in extraction buffer (50 mM HEPES, 50 mM KCl, 300 mM NaCl, 0.1 mM EDTA, 1 mM DTT, 10% glycerol, 0.2 mM NaF, 0.2 mM Na_3_VO_4_, 0.1 mM PMSF) and incubated on ice for 20 min. with agitation. The nuclear proteins in the supernatant were recovered after centrifugation (12 000×*g*, 20 min, 4°C) to remove nuclear debris. Proteins present in extracts were quantified using the Coomassie protein assay (Pierce) referenced against a bovine serum albumin standard and stored in aliquots at –80°C. Extracts were used for western blot analysis using anti-NF-κB p65 antibodies (Santa Cruz, SC372). The quality of the fractionation was confirmed using tubulin as a cytoplasmic marker and lamin A/C as a nuclear marker.

### Transfections and luciferase assays

A549 cells were seeded into 24-well tissue culture plates to get a 40–60% confluence 24 h later. Cells were washed twice with PBS before transfection. Transfection experiments were carried out in 500 µl of Opti-MEM Reduced-Serum Medium (Invitrogen) using Lipofectamine™ 2000 Transfection Reagent following manufacturer's recommendations (Invitrogen).

For luciferase experiments, the PathDetect® NF-κB *cis*-Reporting plasmid (250 ng; Stratagene) or vector containing 5′-flanking region of human BD2 (1 µg; [26]) were co-transfected with the pRL-TK *Renilla* Luciferase control reporter vector (20 ng; Promega). For luciferase assays, cells were lysed with Passive Lysis Buffer (Promega). Luciferase activity was assayed using the Dual Luciferase Assay kit according to the manufacturer's instructions (Promega). Firefly luciferase values were normalized to *Renilla* control values. Results were plotted as Relative luciferase activity compared with activity measured in non stimulated control cells. The luciferase assay was carried out in triplicate on at least three independent occasions.

### Antimicrobial peptide resistance assay

The assay was done as we have recently described [27]. Briefly, bacteria were grown at 37°C in 5 ml LB, harvested (5000×*g*, 15 min, 5°C) in the exponential phase of growth and washed three times with PBS. A suspension containing approximately 10^5^ cfu/ml was prepared in 10 mM PBS (pH 6.5), 1% Tryptone Soya Broth (TSB; Oxoid), 100 mM NaCl. Aliquots (5 µl) of this suspension were mixed in Eppendorf tubes with various concentrations of BD2. In all cases the final volume was 30 µl. After 3 h incubation, the contents of the Eppendorf tubes were 1∶10 diluted with PBS and 100 µl immediately plated on LB agar. Colony counts were determined and results were expressed as percentages of the colony count of bacteria not exposed to antibacterial agents. The 50% inhibitory concentration of BD2 (IC_50_) was defined as the concentration producing a 50% reduction in the colony counts compared with bacteria not exposed to the antibacterial agent.

All experiments were done with duplicate samples on three independent occasions.

### Small interfering RNA (siRNA)

RNA-mediated interference for downregulating TLR4 and TLR2 was done by the transfection of TLR4 siRNA (catalogue #HSS110818) or TLR2 siRNA (catalogue #HSS110813) purchased from Invitrogen. Stealth™ RNAi Negative Control Medium and Low GC were used as control interference RNA for TLR2 and TLR4 respectively. 20 nM of siRNA per well was used for transfection using Lipofectamine™ 2000 Transfection Reagent and following manufacturer's recommendations (Invitrogen). Cells were infected 48 h post-transfection as previously described.

### Real time quantitative PCR (RT-qPCR)

Quantification of mRNA levels was conducted by RT-qPCR. RNA was purified using a Nucleospin RNAII kit (Macherey-Nagel) exactly as recommended by the manufacturer. cDNA was obtained by retrotranscription of 1.5–2 µg of total RNA using a commercial RT^2^ First Strand kit as recommended by the manufacturer (SABioscience). The reaction included one step to eliminate traces of genomic DNA. Real-time PCR (RT-PCR) analyses were performed with a Smart Cycler real-time PCR instrument (Cepheid, Sunnyvale, CA). To amplify human TLR2 and TLR4, 500 ng of cDNA were used as a template in a 25-µl reaction containing 1 x Quantitect SYBR Green PCR mix (Qiagen) and QuantiTect Primer Assays [Qiagen; catalog numbers QT00236131 (TLR2) and QT00035238 (TLR4)]. As internal control we amplified the human housekeeping *GAPDH* using 50 ng of cDNA and the following intron spanning primers: (sense, 5′- GAAGGTGAAGGTCGGAGTC-3′; antisense, 5′- GAAGATGGTGATGGGATTTC-3′). For detection of *TLR2* and *TLR4*, the thermocycling protocol was as follows: 95°C for 15 min for hot-start polymerase activation, followed by 45 cycles of denaturation at 95°C for 30 sec, annealing at 60°C for *TLRs* or 54°C for *GADPH* for 30 sec; and extension at 72°C for 30 sec. The SYBR Green dye was measured at 521 nm during the annealing phase. The threshold cycle (Ct) value reflects the cycle number at which the fluorescence generated within a reaction crosses a given threshold. The Ct value assigned to each well thus reflects the point during the reaction at which a sufficient number of amplicons have been accumulated. The relative mRNA amount in each sample was calculated based on its Ct in comparison with the Ct of GAPDH. Specificity of the PCR products was determined by melting curve analysis and amplification products were resolved on a 1.5% agarose gel to confirm the correct size of the amplicons (92, 102 and 226 bp for *TLR2*, *TLR4* and *GAPDH* respectively).

### LPS purification

*A. baumannii* ATCC 17978 LPS was purified from 10 mg of lyophilized bacterial cells using the Tri-Reagent extraction method as previously described [28]. LPS was further purified by DNAsa, RNAase and proteinase K treatments [29] before precipitation with 0.375 M magnesium chloride in 95% ethanol. The pellet was resuspended in water and lyophilized. This LPS did not activate the NF-κB dependent luciferase reported gene in HEK293 cells transiently transfected with human TLR2 (for a description of the assay see [30]).

### Method for lipid A extraction

*A. baumannii* ATCC 17978 lipid A was extracted using an ammonium hydroxide/isobutyric acid method [31] and subjected to negative ion matrix-assisted laser desorption ionization time-of-flight (MALDI-TOF) mass spectrometry analysis. Briefly, lyophilized cells (10 mg) were resuspended in 400 µl isobutyric acid/1M ammonium hydroxide (5∶3, v/v) and incubated in a screw-cap test tube at 100°C for 2 h, with occasional vortexing. Samples were cooled in ice water and centrifuged (2,000×*g* for 15 min). The supernatant was transferred to a new tube, diluted with an equal volume of water, and lyophilized. The sample was washed twice with 400 µl methanol and centrifuged (2,000×*g* for 15 min). The insoluble lipid A was solubilized in 100–200 µl chloroform/methanol/water (3∶1.5∶0.25, v/v/v). Analyses were performed on a Bruker Autoflex II MALDI-TOF mass spectrometer (Bruker Daltonics, Incorporated) in negative reflective mode with delayed extraction. Each spectrum was an average of 300 shots. A peptide calibration standard (Bruker Daltonics) was used to calibrate the MALDI-TOF. The ion-accelerating voltage was set at 20 kV. Dihydroxybenzoic acid (Sigma Chemical Co., St. Louis, MO) was used as a matrix. A few microliters of lipid A suspension (1 mg/ml) were desalted with a few grains of ion-exchange resin (Dowex 50W-X8; H^+^) in an Eppendorf tube. A 1 µl aliquot of the suspension (50–100 µl) was deposited on the target and covered with the same amount of the matrix suspended at 10 mg/ml in a 0.1 M solution of citric acid.

### Statistical methods

Results are expressed as mean ± SD. Differences between experimental groups were analyzed by non-parametric two sided Mann Whitney *U*-test or one-way analysis of variance using Kruskal-Wallis contrasts as appropriate (GraphPad Sotware Inc.). A p value lower than 0.05 was considered significant.

## Results

### *A. baumannii* induced the expression of IL-8 and β-defensin 2 by human airway epithelial cells

**Figure 1:**
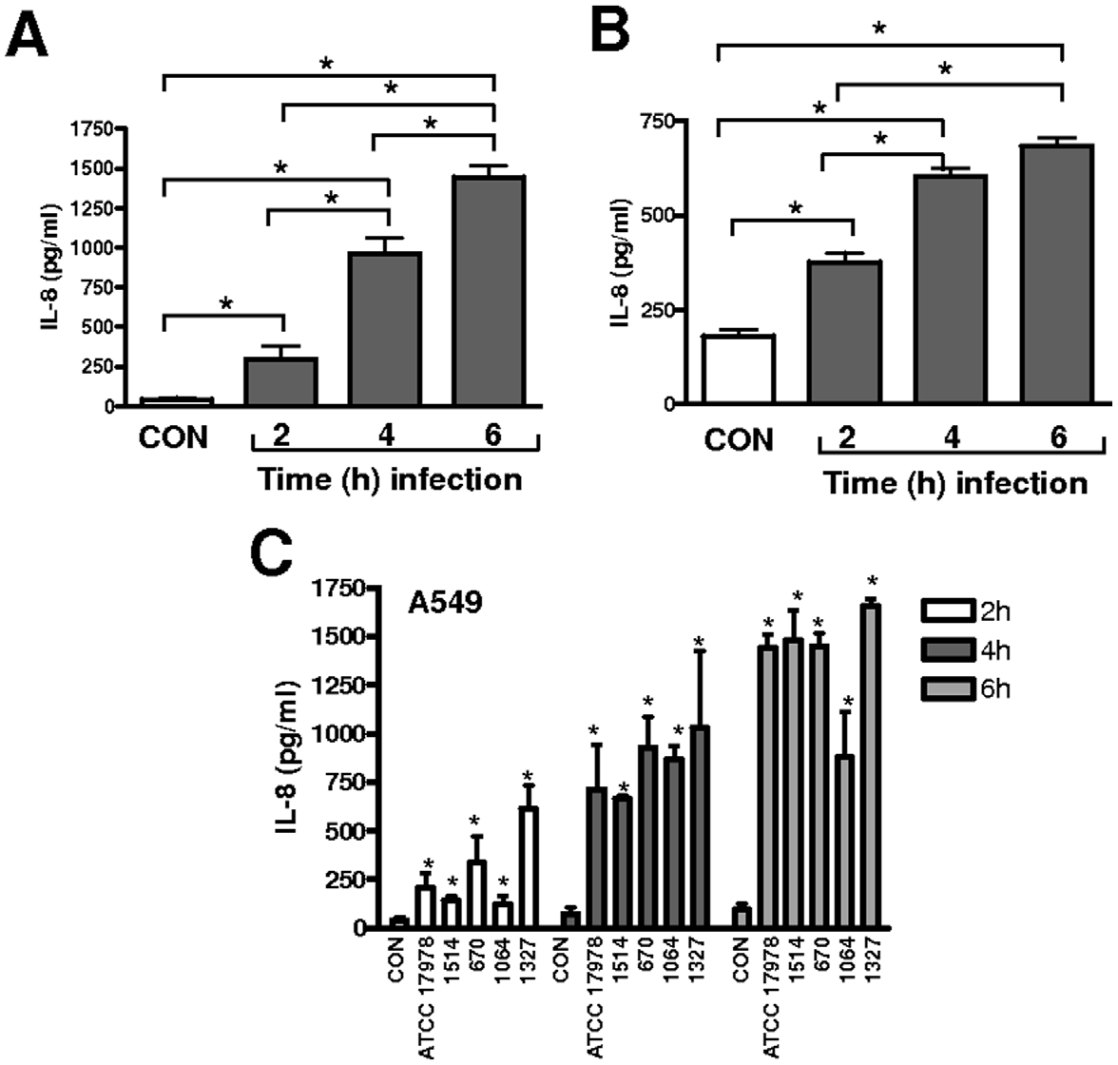
*Acinetobacter baumannii* induces the secretion of IL-8 by airway epithelial cells. (A–B) ELISA of IL-8 released by A549 (panel A) or NHBE (panel B) cells left untreated (CON) or infected for different time points with *A. baumannii* ATCC 17978 (n = 3). *, *P*<0.05 (for the indicated comparisons; one-way ANOVA). (C) ELISA of IL-8 released by A549 left untreated (CON) or infected for different time points with different *A. baumannii* strains (n = 3). *, *P*<0.05 (results are significantly different from the results for untreated cells; one-way ANOVA).

We assessed whether A. *baumannii* induces the expression of IL-8 by human airway epithelial cells. Time-course experiments showed that there was a correlation between the duration of the infection with *A. baumannii* strain ATCC 17978 and the levels of IL-8 secreted by A549 (Figure 1A) and NHBE cells (Figure 1B). The widespread number of *A. baumannii* strains resistant to antibiotics led us to explore whether the increased expression of IL-8 by infected cells is a general theme for *A. baumannii*. All the strains tested induced the secretion of IL-8 by A549 cells and there was a correlation between duration of the infection and the secretion of IL-8 (Figure 1C). No significant differences were found in the levels of IL-8 induced by the strains at any time point (Figure 1C).

**Figure 2:**
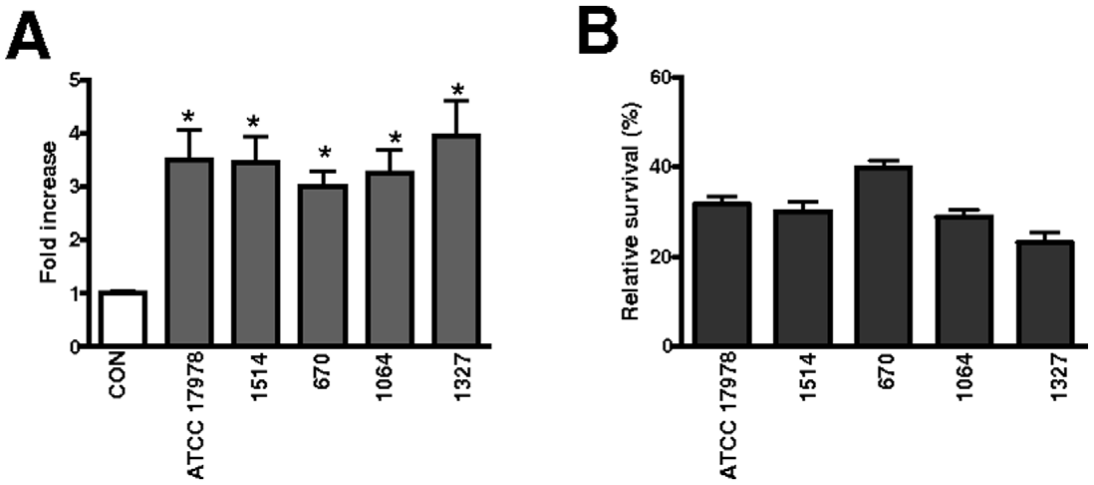
*Acinetobacter baumannii* up-regulates the expression of human β-defensin 2 (BD2) and it is susceptible to this defensin. (A) A549 cells were transfected with BD2 luciferase reporter gene and *Renilla* luciferase plasmid. Cells were infected with *A. baummannii* strains and BD2 promoter activation was measured 6 h post-infection. Activity is normalized by correction of *Renilla* expression and is presented relative to the cells untreated. CON, non infected cells. Bars represent mean ± SD (n = 3) *, results are significantly different (*P*<0.05; one-tailed *t* test) from the results for non infected cells (CON). (B) Survival of bacteria (percentage of cell colonies with respect to not exposed to agents) in presence of 3 µg/ml of BD2. Error bars display standard deviation from the mean of three experiments, each one run in duplicate.

Airway epithelial cells up-regulate the expression of BD2 upon infection [15]–[17]. We sought to determine whether *A. baumannii* increases the expression of BD2 in A549 cells. Cells were transiently transfected with a luciferase reporter construct controlled by the promoter region of BD2. This plasmid has been used previously to monitor the expression of BD2 in airway epithelial cells [26]. BD2-dependent luciferase activity was induced by all *A. baumannii* strains tested (Figure 2A). Survival assays shown in Figure 2B demonstrated that *A. baumannii* strains were susceptible to BD2 (final concentration of 3 µg/ml) and no significant differences were found between strains. To further confirm these findings, we performed dose-response experiments which allowed us to determine the 50% inhibitory concentration (IC_50_) of BD2 for these strains. IC_50_ of BD2 for strain ATCC 17978 was 1.51±0.03 µg/ml, which was similar to those found for strains 1514, 670, 1064 and 1327 (1.85±0.05 µg/ml, 1.01±0.04 µg/ml; 1.34±0.03 µg/ml and 1.65±0.06 µg/ml respectively; p>0.05 for all comparisons).

### Activation of NF-κB and MAPKs signalling pathways is required for *A. baumannii*-induced IL-8 expression

**Figure 3:**
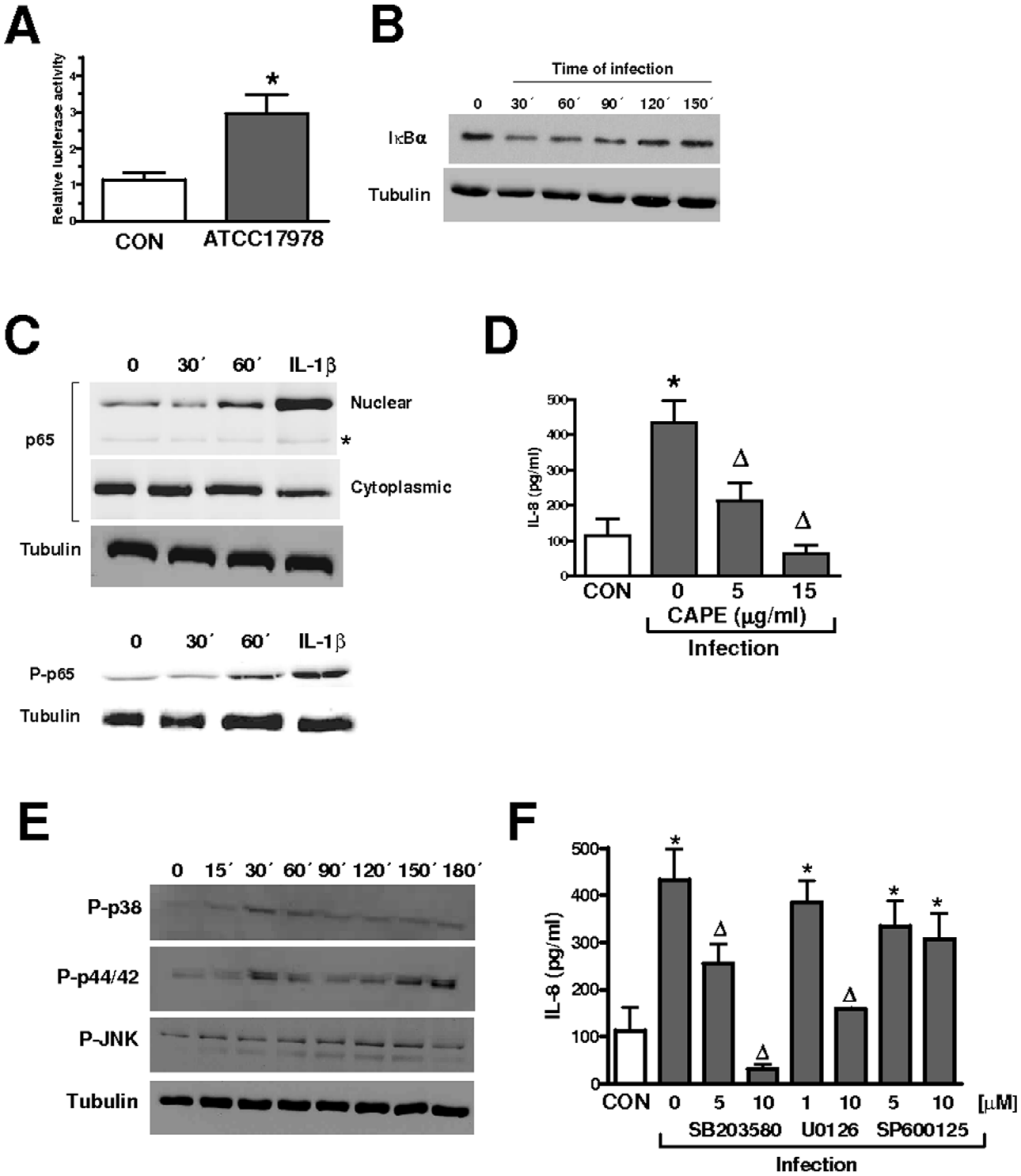
Activation of NF-κB and MAPKs is required for A*cinetobacter baumannii*-induced IL-8 expression. (A) Activation of a NF-κB luciferase reporter plasmid in A549 cells left non treated (CON) or infected for 6 h with *A. baumannii* ATCC 17978. Activity is normalized by correction of *Renilla* expression and is presented relative to the cells untreated (the data are means and SD.; n = 3). (B) Immunoblot analysis of IκBα levels in lysates of A549 cells left untreated (time 0) or infected for different time points with *A. baumannii* ATCC 17978. Data are representative of three independent experiments. (C) Upper panels, immunoblots showing p65 levels in nuclear and cytoplasmic extracts of A549 cells infected with *A. baumannii* ATCC 17978 for different time points. Asterisk denotes a protein recognized by the antibody which is only present in nuclear extracts and also serves as a loading control. Blots of cytoplasmic extracts were reprobed with polyclonal antibody anti human tubulin to control that equal amounts of proteins were loaded in each lane. Lower panels, immunoblots showing phospho-p65 and tubulin levels in lysates of A549 infected with *A. baumannii* ATCC 17978 for different time points. IL-1β (20 ng/ml for 30 min) was used as a positive control for NF-κB activation. The results are representative of three independent experiments. (D) ELISA of IL-8 released by A549 cells left untreated (CON, white bar) or infected for 4 h with *A. baumannii* ATCC 17978 in the absence or presence of different concentrations of CAPE, an inhibitor of NF-κB, which was added 1 h before infecting the cells (n = 3). (E) Immunoblots showing phospho-p38 (P-p38), phospho p44/42 (P-p44/42), phospho JNK (P-JNK) The results are representative of three independent experiments. (F) ELISA of IL-8 released by A549 cells left untreated (CON, white bar) or infected for 4 h with *A. baumannii* ATCC 17978 in the absence or presence of different concentrations of SB203580 (5 and 10 µM; p38 MAPK inhibitor), U0126 (1 and 10 µM; p44/42 MAPK inhibitor) or SP600125 (5 and 10 µM; JNK MAPK inhibitor) which were added 2 h before infecting the cells (n = 3). *, *P*<0.05 (results are significantly different from the results for untreated cells; one-way ANOVA). Δ, *P*<0.05 (results are significantly different from the results for infected cells in the absence of inhibitor).

Given that several studies show that NF-κB activation is associated with IL-8 expression [10], we sought to determine whether *A. baumannii* activates this transcriptional factor. A549 cells were transiently transfected with a NF-κB-dependent luciferase reporter followed by infection and *A. baumannii*-induced NF-κB activation was measured as relative luciferase activity. NF-κB-dependent luciferase acitivity was induced upon infection with *A. baumannii* (Figure 3A). Upon cellular stimulation, in the canonical pathway of NF-κB activation IκBα, present in the cytosol complexed to NF-κB dimers preventing nuclear translocation, becomes phosphorylated which leads to ubiquitination of the protein and its subsequent degradation by the proteosome thereby allowing nuclear translocation of NF-κB, process also linked to phosphorylation of the p65 subunit [32]. Therefore we analyzed the levels of IκBα in cytoplasmic extracts of infected cells by immunoblot. IκBα degradation was apparent 30 min post infection whereas 120 min post infection the IκBα levels were similar to those of non infected cells (Figure 3B). Concomitantly, *A. baumannii* induced the nuclear translocation of NF-κB and the phosphorylation of the p65 subunit (Figure 3C). To determine the contribution of NF-κB activation to *A. baumannii*-induced IL-8 expression, we asked whether CAPE, a chemical inhibitor used to block NF-κB signaling pathway [33], alters *A. baumannii*-induced IL-8 expression. As shown in Figure 3D, CAPE reduced *A. baumannnii*-triggered IL-8 levels. Control experiments showed that addition of dimethyl sulfoxide (DMSO) (the vehicle solution used for CAPE) to *Acinetobacter*-infected cells did not affect IL-8 levels (pg/ml in the absence of DMSO 433±65; pg/ml in the presence of DMSO 505±95). CAPE-treated cells (15 µg/ml) expressed levels of IL-8 (95±85 pg/ml) similar to those of non-treated cells (113±50 pg/ml). Collectively, these data demonstrated that IκBα-dependent activation of NF-κB is required for *A. baumannii* induction of IL-8 expression in A549 cells.

MAPKs are important regulators of pro-inflammatory gene expression including IL-8 [10]. We asked whether MAPKs are involved in A. *baumannii*-induced IL-8 expression. MAPKs p38, p44/42 and JNK activations occur through phosphorylation of serine and threonine residues. Western blot analysis revealed that *A. baumannii* induced activation of the three MAPKs (Figure 3E). To explore whether the activation of MAPKs is involved in *A. baumannii*-induced IL-8 expression, the infections were carried out in the presence of chemical inhibitors for each MAPK. Figure 3F shows that SB203580, a specific inhibitor of p38 MAPK, reduced *A. baumannii*-induced IL-8 expression. The p44/42 inhibitor, U0126, also reduced *A. baumannii*-dependent IL-8 expression but only at the highest dose tested (Figure 3F). Finally, SP600125, a JNK inhibitor, did not alter *A. baumannii*-induced IL-8 expression (Figure 3F). SB203580, U0126 and SP600125-treated cells secreted similar amounts of IL-8 (120±95; 95±67 and 135±87 pg/ml, respectively) than non-treated cells (130±75 pg/ml). Taken together, these findings indicate that activation of MAPKs p38 and p44/42 is involved in *A. baumannii*-induced IL-8 expression in A549 cells.

### *A. baumannii*-induced IL-8 expression is dependent on TLR activation

**Figure 4:**
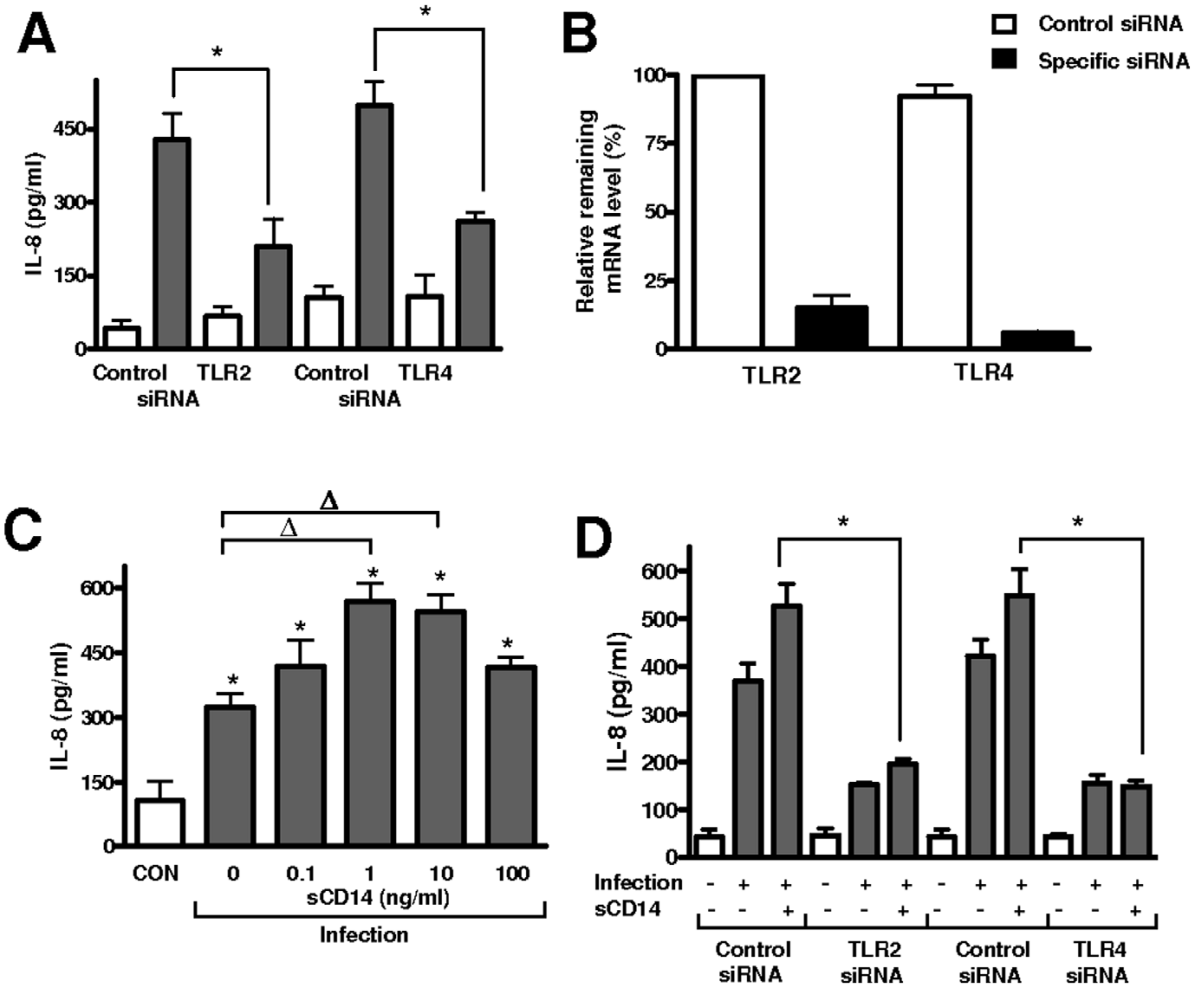
Role of TLR2, TLR4 and sCD14 in *Acinetobacter baumannii*-induced IL-8 expression. (A) ELISA of IL-8 secreted by A549 cells transfected with either control, TLR2, or TLR4 siRNAs, which were left untreated (white bars) or infected for 4 h with *A. baumannii* ATCC 17978 (data are means and SD.; n = 3). *, *P*<0.05 (one-way ANOVA). (B) siRNA efficiency was quantified by RT-qPCR in samples from the same experiment shown in panel A. mRNA level was normalized to GADPH and then relative mRNA levels in cells transfected with control siRNA or specific siRNA were compared. mRNA levels in cells transfected with control siRNA were set to 100% (the data are means and SD.; n = 3). (C) ELISA of IL-8 secreted by A549 left untreated (CON, white bar) or infected with *A. baumannii* ATCC 17978 for 4 h in the absence or presence of different amounts of sCD14 (n = 3). *, *P*<0.05 (results are significantly different from the results for untreated cells; one-way ANOVA). Δ, *P*<0.05 (results are significantly different from the results for infected cells in the absence of sCD14). (D) ELISA of IL-8 secreted by A549 cells transfected with either control, TLR2, or TLR4 siRNAs, which were left untreated (white bars) or infected for 4 h with *A. baumannii* ATCC 17978 in the presence or absence of sCD14 (1 ng/ml) (data are means and SD.; n = 3). *, *P*<0.05 (one-way ANOVA).

In most cases, the infection-dependent activation of NF-κB and MAPKs signaling pathways is due to the engagement of TLRs [34], [35]. Therefore it could be predicted that TLR-dependent signaling would be important in the recognition of *A. baumannii* by A549 cells. To assess whether TLRs are involved in *A. baumannii*-induced IL-8 expression, siRNA was used to knock-down TLR2 or TLR4 expression in A549 cells. As shown in Figure 4A, in TLR2 knock-down cells, *A. baumannii* induced significantly less IL-8 than in control cells. A decreased in the levels of IL-8 was also observed in *A. baumannii*-infected TLR4 knock-down cells in comparison to control cells (Figure 4A). The efficiency of siRNA-mediated downregulation of target gene mRNA levels was confirmed by RT-qPCR (Figure 4B).

CD14 is a 55-kDa GPI-linked glycoprotein which also participates in pathogen recognition and uses TLRs as co-receptors in signal transduction [36]. We and others have previously demonstrated that A549 cells do not express CD14 on their surface [25], [37], [38]. Host cells lacking CD14 on their surface can compensate its absence with soluble CD14 (sCD14) [39]–[41]. To test the effect of CD14 on the activation of A549 cells, recombinant human sCD14 was added to the culture medium. When infections were performed in the presence of sCD14, the secretion of IL-8 peaked when 1 ng/ml sCD14 was used (Figure 4C). Higher doses of sCD14 did not further increase *A. baumannii*-induced IL-8 levels. In fact, IL-8 levels were similar between *Acinetobacter*-infected cells with and without 100 ng/ml sCD14 (one-way ANOVA; p>0.05). Control experiments showed that sCD14 alone, even at the highest dose tested, did not induce the secretion of IL-8 by A549 cells (data not shown). In order to explore the role of TLRs in sCD14-mediated signal transduction, TLR2 or TLR4 were knocked down by siRNA and infections were done in the presence of sCD14 (1 ng/ml). Results shown in Figure 4D demonstrate that sCD14 did not increase *A. baumannii*-induced IL-8 secretion in either TLR2 or TLR4 knock-down cells.

In summary, these data indicate that the recognition of *A. baumannii* by A549 cells involved TLR2 and TLR4 and that A549 cells could use sCD14 as TLR co-receptor to detect *A. baumannii*.

### *A. baumannii* LPS induced the expression of IL-8 by human airway epithelial cells

**Figure 5:**
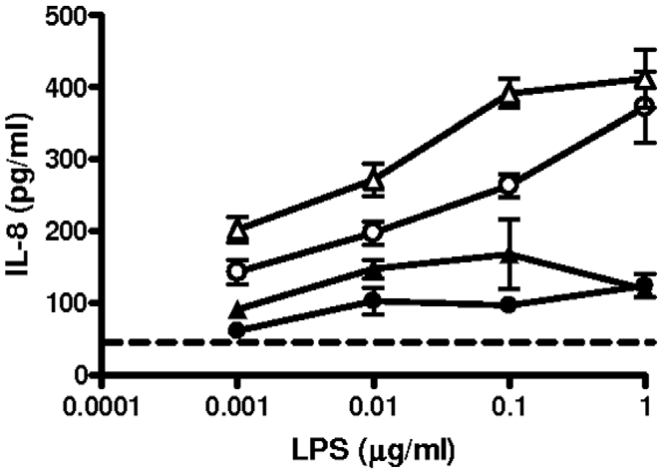
*Acinetobacter baumannii* and *Escherichia coli* LPSs-induced IL-8 from airway epithelial cells. A549 cells were challenged with medium alone (dashed line) or with 0.001–1 µg/ml of each LPS preparation in the absence (black symbols) or presence (white symbols) of sCD14 (1 ng/ml). Supernatant IL-8 levels were measured at 18 h. Results are presented as means and SD.; (n = 3). ▵, ▴ *A. baumannii* LPS; ○, • *E. coli* LPS.

We aim to investigate the inflammatory potential of *A. baumannii* LPS in airway epithelial cells in comparison to *Escherichia. coli* LPS. Both LPSs induced the secretion of IL-8 by A549 cells being 0.01 µg/ml the amount of both LPSs which significantly stimulated the secretion of IL-8 (one-way ANOVA; p<0.05) (Figure 5). When stimulations were done in the presence of sCD14 (1 ng/ml), a significant increase in the levels of IL-8 was found for both LPSs (one-way ANOVA; p<0.05). However, the effect was more dramatic for *A. baumannii* LPS than for *E. coli* one.

### *A. baumannii* lipid A analysis

**Figure 6:**
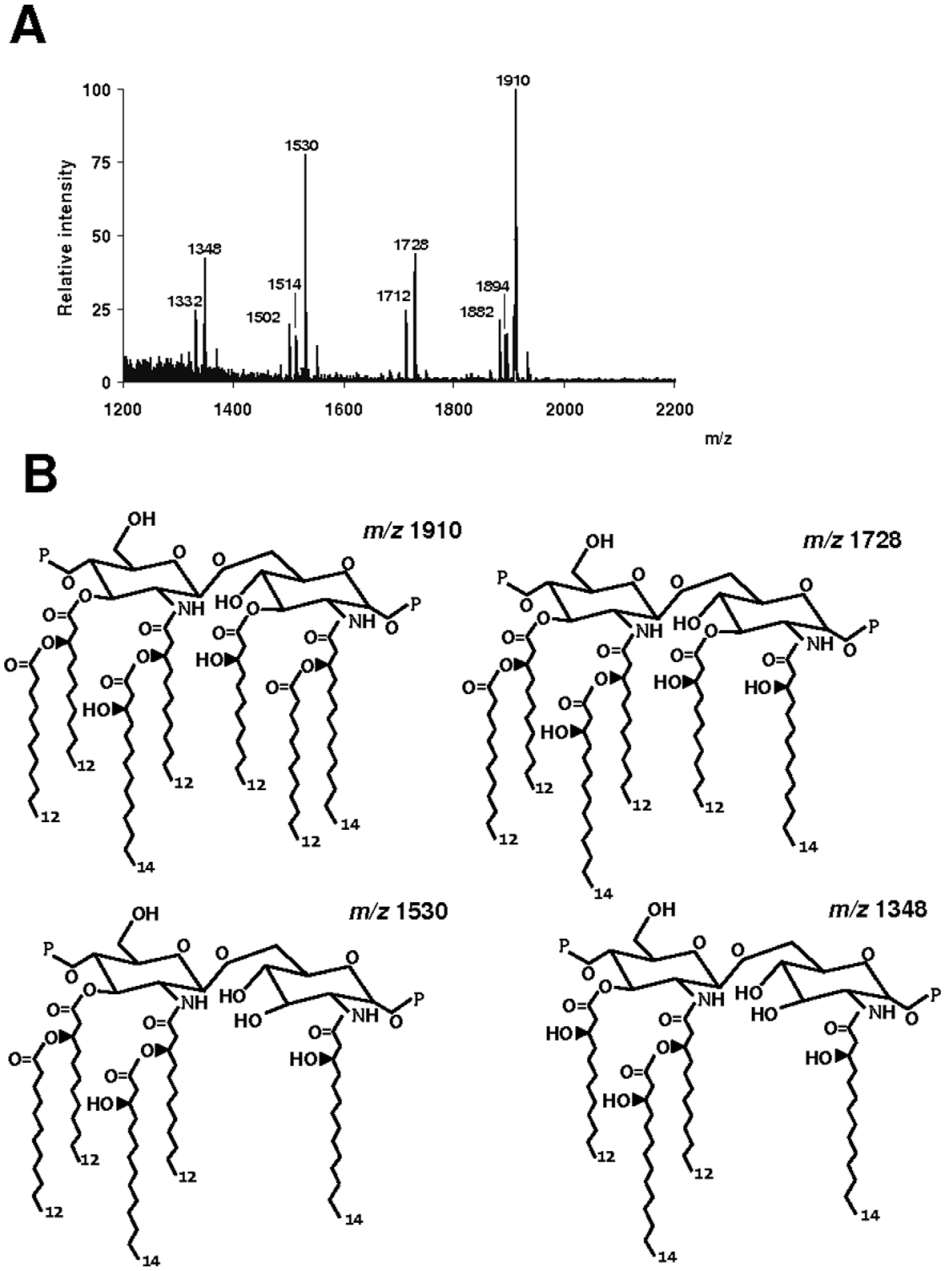
Analysis of *Acinetobacter baumannii* lipid A. (A) Negative ion MALDI-TOF mass spectra of lipid A isolated from *Acinetobacter baumannii* ATCC 17978. (B) Proposed structures of the main molecular species present in *Acinetobacter baumannii* ATCC 17978 lipid A.

Given that the LPS lipid A moiety is the part of molecule responsible for its bioactivity, we characterized the lipid A moiety of strain ATCC 17978 by MALDI-TOF mass spectrometry. Previous studies determined unambiguously that lipid A disaccharide backbone of *Acinetobacter* species is composed of two 2-amino-2-deoxyglucose residues (GlcNI and GlcNII) linked by a β-(1-6) glycosidic linkage and phosphorylated at positions 1 and 4′ [42]–[44]. In another hand, the lipid A structure of *A. radioresistens* has been recently studied by MALDI-TOF. This species contained predominantly hepta-acylated lipid A containing two phosphates, two 2-amino-2-deoxyglucose residues substituted with either four 12:0(3-OH) and three 12:0, or three 12:0(3-OH), three 12:0 and one 14:0(3-OH) [43]. Taken into account that a wealth of evidence indicates that lipid A architecture is conserved within a genus [45]; we expected that *A. baumannii* would contain a lipid A structure similar to that from *A. radioresistens,* perhaps with minor structural modifications related to the type of acylation. Analysis of the negative ion mass spectrum of the intact lipid A fraction of strain ATCC 17978 revealed a major ion peak *m/z* 1,910 with two minor associated peaks *m/z* 1,894 (Δ*m/z* 16) and *m/z* 1,882 (Δ*m/z* 28) attributable to certain microheterogenicity of the sample. Proposed structure corresponding to *m/z* 1,910 would correspond to a hepta-acylated lipid A with two 2-amino-2-deoxyglucose residues, two phosphates, three 12:0(3-OH), two 14:0(3-OH) and two 12:0 (Fig. 6A and B). Δ*m/z* 16 indicates a non-stoichiometric substitution of one 14:0(3-OH) with one 14:0. *m/z* 1,882 (Δ*m/z* 28) may represent four 12:0(3-OH), two 12:0 and one 14:0(3-OH). Molecular species at *m/z* 1,728 and 1,712, corresponding to a hexa-acylated lipid A, differ from *m/z* 1,910 and *m/z* 1,894 species by missing 12:0 respectively (Figure 6A and B). Ion peak *m/z* 1,530 may represent a penta-acylated lipid A lacking one 12:0 whereas molecular species *m/z* 1,348 may correspond to a tetra-acyl lipid A containing two 2-amino-2-deoxyglucose residues, two phosphates, two 12:0(3-OH) and two 14:0(3-OH) (Figure 6A and B). Analysis of the negative ion mass spectrum of the intact lipid A fraction of *A. baumannii* strains 1514, 670, 1064 and 1327 (Figure S1) revealed that lipid As of the four strains contained the major ion peaks *m/z* 1,910 and *m/z* 1,728 found in lipid A fraction of strain ATCC 17978 whereas minor ion peaks *m/z* 1,530 and *m/z* 1,348 were also present in lipid A from strains 1514, 670 and 1327 but absent in strain 1064.

Altogether, these data indicate that the lipid A fraction of *A. baumannii* strains, including multidrug resistant ones, would contain two major species corresponding to a hepta-acylated lipid A with two 2-amino-2-deoxyglucose residues, two phosphates, three 12:0(3-OH), two 14:0(3-OH) and two 12:0 (*m/z* 1,910) or a hexa-acylated lipid A with two 2-amino-2-deoxyglucose residues, two phosphates, three 12:0(3-OH), two 14:0(3-OH) and one 12:0 (*m/z* 1,728).

## Discussion

In this work, we demonstrate that *A. baumannii* infection of airway epithelial cells results in the secretion of IL-8 and up-regulation of BD2 expression. Mechanistically, we demonstrate that *A. baumannii*-triggered IL-8 depends on the activation of NF-κB, and MAPKs p38 and p44/42. In addition, we show that *A. baumannii* engages TLR2 and TLR4 to activate the expression of IL-8 and that sCD14 contributes to the recognition of this pathogen.

Neutrophil infiltration is a hallmark of the initial acute inflammatory response. In humans, IL-8 has a prominent role in the recruitment and activation of neutrophils [10]. Animal experiments have revealed that the predominantly neutrophilic inflammation in the airways of infected mice is essential for the eradication of *A. baumannii* from the lungs [22]–[24]. Furthermore, clinical studies have shown that *A. baumannii* is a common isolate in neutropenic febrile patients [46]. Therefore, our data showing that *A. baumannii*-stimulated airway epithelial cells secrete high levels of IL-8 are consistent with the idea that an important function of airway epithelial cells is to initiate the lung influx of neutrophils. All *A. baumannii* strains tested in this study, including multidrug resistant clinical isolates, induced similar levels of IL-8 thereby suggesting that this feature could be a general theme of *A. baumannii*. It is then tempting to speculate that treatments designed to boost this cell response would help to clear *A. baumannii* infections, even those caused by multidrug resistant strains. Giving support to this idea, in a mouse model of pneumonia, administration of MIP-2, a functional mouse homologue of IL-8, significantly enhanced the clearance of *A. baumannii* from the lungs [23], [24].

BDs are known to play an important role in lung innate immune response due to their potent activity against gram negative and positive bacteria, fungi, and viruses. However, BDs not only protect the lung against invading microbes, but they also modulate the host immune response by providing an interface between innate and adaptive immune response [47]–[50]. Because inflammatory cytokines and pathogens induce the expression of BD2 [14]–[17], it is accepted that this defensin plays an important role in host defence as inducible component of the epithelial barrier. In the present study, we have shown that *A. baumannii* strains not only up-regulated the expression of BD2 but also they were susceptible to it. Therefore, these results demonstrate the capacity of airway epithelial cells to produce mediators necessary for the clearance of *A. baumannii*.

Our results revealed that *A. baumannii* activates NF-κB and MAPKs signaling pathways in airway epithelial cells. Our findings add further evidence to the hypothesis that epithelial cells lining mucosal surfaces orchestrate a defense response upon pathogen challenge by activating NF-κB and MAPKs-dependent signaling pathways [51]. Although p38 and p44/42 MAPKs were required for *A. baumannii*-induced IL-8, the pathogen also activated JNK MAPK hence suggesting that there are other host cell responses activated in a JNK MAPK-dependent manner. Future studies will aim to identify these responses.

Accumulating evidence exist showing that engagement of TLRs triggers the activation of NF-κB and MAPKs and subsequently the production of cytokines and chemokines [52]. Therefore, we speculated that activation of airway epithelial cells by *A. baumannii* could involve TLRs-dependent signaling pathways. Our results showed that both TLR2 and TLR4 play an important role in the production of IL-8 by *A. baumannii* infected A549 cells. These data are in partial agreement with the results reported by Knapp and co-workers [22] that indicate that only TLR4-dependent signaling plays an important role in sensing of *A. baumannii in vivo* because TLR4-deficient mice displayed an impaired lung inflammatory response upon *A. baumannii* infection. In contrast, TLR2-deficient mice displayed an increased inflammatory response associated with accelerated elimination of the pathogen from the lung. It should be noted that *in vivo* scenario is quite complex and the final outcome of the infection is dependent on the concerted action of several cell types such as epithelial cells, alveolar macrophages, neutrophils and lymphocytes, all of them expressing TLR2, recruited and/or activated upon infection whereas here we have just tested the interplay between *A. baumannii* and airway epithelial cells. In addition, the discrepancy between our data and those obtained *in vivo* may be also attributable to differences between mice and humans related to TLR2-dependent recognition of *Acinetobacter* and the cross-talks between different signaling cascades.

To further characterize the receptors involved in the generation of *A. baumannii*-mediated responses, we evaluated the role of CD14 which may act as co-receptor for bacterial recognition in epithelial cells [36]. Our results showed that A549 cells could use sCD14 as TLR co-receptor to recognize *A. baumannii*. Supporting that sCD14 is present in the airways, the molecule has been detected in bronchoalveolar fluid of healthy subjects [53] and we have demonstrated that epithelial cells may serve as one possible source of sCD14 because airway epithelial cells primed with inflammatory cytokines release sCD14 [54].

Our data demonstrated that *A. baumannii* LPS stimulates airway epithelial cells to secrete IL-8 thereby suggesting that this LPS-elicited IL-8 could induce a pulmonary influx of neutrophils. Supporting this, Knapp and colleagues [22] showed that intranasal administrated *A. baumannii* LPS indeed triggers the recruitment of neutrophils to the lungs. The ability of LPSs to evoke inflammatory responses and the potency of them are directly related to the structure of the molecule which in turn affects the interaction with the LPS receptor complex. Our structural analysis of the lipid A moieties of five *A. baumannii* strains by MALDI-TOF mass spectrometry revealed that *A. baumannii* lipid A is a bisphosphorylated diglucosamine to which are attached at least six saturated fatty acyl chains with lengths of 12 or 14 carbons. This structure matches with the so-called “canonical lipid A structure” which is associated with the highest level of activation of the human immune system [55]. This maybe the molecular explanation underlying the unusually severe systemic inflammatory reaction in response to *A. baumanniii* infection found in some individuals [56], [57].

It is worth commenting on the clinical implications of our findings. The global emergence of *A. baumannii* strains resistant to virtually all antibiotics makes it urgent to develop effective therapeutics based on new targets. Our findings together with those obtained *in vivo* [22]–[24] argue in favour of therapies based on stimulation of TLR-dependent pathways in airway epithelial cells thereby leading to chemokine secretion and the subsequent recruitment of neutrophils. However, due to the high entodotoxic potential of *A. baumannii* LPS care should be taken to induce an overwhelming and detrimental inflammatory response.

## Supporting Information

Figure S1 Negative ion MALDI-TOF mass spectra of lipid As isolated from Acinetobacter baumannii strains 670, 1514, 1327 and 1064.
(6.53 MB TIF)
