# Supplementary figures and images for: Dissection of Host Cell Signal Transduction during Acinetobacter baumannii – Triggered Inflammatory Response
Source: PLoS One. 2010 Apr 7;5(4):e10033. doi: 10.1371/journal.pone.0010033 (PMC2850920; doi:10.1371/journal.pone.0010033)

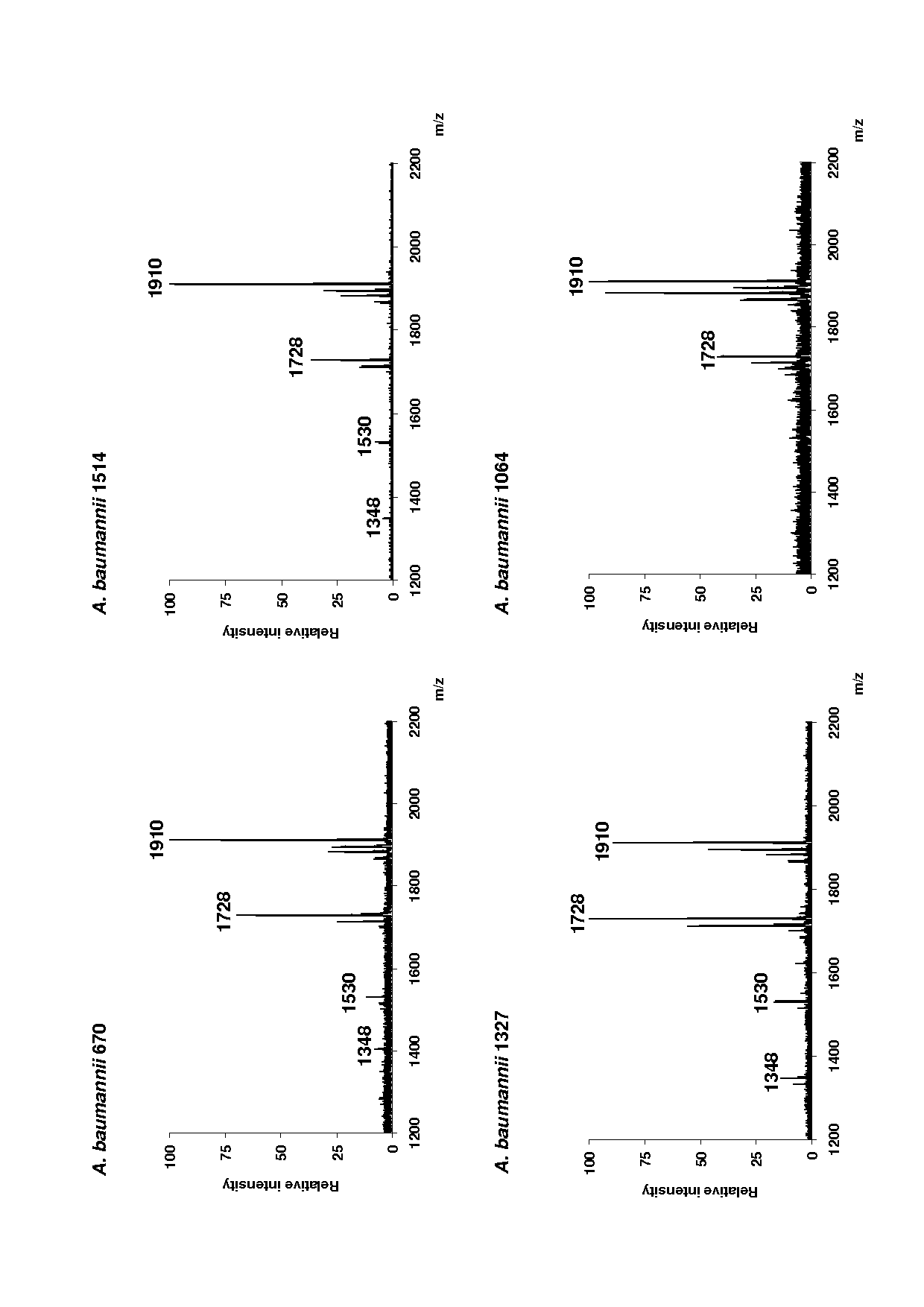

Supplement: Figure S1 — Negative ion MALDI-TOF mass spectra of lipid As isolated from Acinetobacter baumannii strains 670, 1514, 1327 and 1064. (6.53 MB TIF) [file pone.0010033.s001.tif]
